# Non-occupational exposure to heavy metals of the residents of an industrial area and biomonitoring

**DOI:** 10.1007/s10661-016-5693-5

**Published:** 2016-11-16

**Authors:** Luigi Vimercati, Antonio Baldassarre, Maria F Gatti, Tommaso Gagliardi, Maria Serinelli, Luigi De Maria, Antonio Caputi, Angelica A Dirodi, Ida Galise, Francesco Cuccaro, Giorgio Assennato

**Affiliations:** 1 Interdisciplinary Department of Medicine, Occupational Medicine “B. Ramazzini”, University of Bari Medical School, 70124 Bari, Italy; 2 ARPA PUGLIA, Environmental Protection Agency of Apulia, Corso Trieste 27, 70126 Bari, Italy; 3 Health Local Unit of Barletta-Andria-Trani, 76121 Barletta, Italy

**Keywords:** Heavy metals, Environmental exposure, Biological monitoring, Resident population, Polluted area

## Abstract

In areas at high environmental risk, a major issue is the assessment of the exposure of the general population to industrial pollutants. To date, few studies have investigated exposure to heavy metals in a population residing in a high risk environmental area. The aim of this study is to evaluate the exposure to heavy metals in the industrial area of Taranto, Southern Italy, through biological monitoring techniques. We measured the levels of inorganic arsenic and methylated metabolites, lead, cadmium, chromium, and manganese in the urine samples of 279 subjects residing in Taranto and neighboring areas. After obtaining informed consent from each participant, qualified health staff administered a standardized structured questionnaire investigating lifestyle habits and assessing any confounding factors. The biological monitoring data showed high urinary concentrations of nearly all of the heavy metals investigated. These findings could be related to the presence of industrial plants and is sufficient to warrant the expectation that local and national institutions should be required to adopt preventive measures to reduce the environmental exposure of the general population to heavy metals.

## Background

The problem of pollution from industrial plant emissions is a highly relevant topic in Italy. Among the main areas affected is Taranto (Southern Italy), the site of a large integrated cycle steel foundry, a refinery, and a cement factory. Many years have passed since the World Health Organization (WHO) first included the Taranto area among those at high environmental risk and highlighted the increased mortality rates, compared to Italy as a whole, for lung, bladder, and liver cancer, as well as cancer of the pleura and non-Hodgkin lymphoma (WHO 1997). Subsequent studies on incident cases of these diseases in Taranto confirmed the existence of a possible link between a higher risk and residing near the source of the harmful emissions (Marinaccio et al. 2011).

A geographic analysis of the tumor death rate in the period 2000–2004 in the various Apulian provinces (Martinelli et al. 2009), based on the Causes of Death Regional Registry data, showed that in the city of Taranto and in the municipalities surrounding the industrial pole, the excess tumor risk ranges between 10 and 13% in both sexes, while for lung cancer, in particular, the excess is 28% in males and 33% in females.

The results of the recent SENTIERI project (Epidemiological Study of Residents in Italian Contaminated Sites), funded by the Italian Ministry of Health and coordinated by National Institutes of Health, showed an excess mortality in all 44 “Sites of National Interest” (SIN) in need of remediation, including Taranto and the nearby Statte municipality (Pirastu et al. 2010; Pirastu et al. 2011).

A later update of SENTIERI focused on only the site of national interest of Taranto, conducting an analysis of mortality data extending from 2003 to 2009 and studying the time trend of mortality from 1980 through 2008, as well as data on tumor incidence in 2006–2007. Overall, these analyses demonstrated an excess mortality in both sexes from causes in which environmental exposure at the SIN plays a defined etiological role or a suspected role based on a priori assessments of the epidemiological evidence (Comba et al. 2012; Pirastu et al. 2013). These results were also confirmed in the latest SENTIERI update, which noted an excess mortality (updated to 2010), cancer incidence (period 1996–2005), and hospital stay (period 2005–2010) in both sexes compared to the expected values (Pirastu et al. 2014).

Environmental monitoring studies and measurements of the industrial emissions in the Ionian-Taranto area have confirmed the existence of environmental pollution (Di Filippo et al. 2005; Ferri et al. 2003; Giua et al. 2005; Liberti et al. 2006; Viviano et al. 2005), supporting the presence of a risk not only for workers directly exposed to the industrial toxic agents but also for the general population, who could suffer exposure from the air, water, and soil (EC-JRC-IPTS 2013).

In particular, the importance of investigating the general public’s exposure to heavy metals lies in their ubiquitous nature since they are also widely distributed in nature, as well as in their harmful effects on human health. Some specific elements (As, Cd, Cr, Ni) are recognized as certainly carcinogenic in humans (IARC 2012a, b), while others such as Hg, Mn, and Pb can have adverse effects on various organs and systems (Flora et al. 2012; Guilarte 2011; Rice et al. 2014).

Previous studies investigating the exposure of the general population and of industrial workers to heavy metals in the Ionian-Taranto area did not find significant differences in the urinary excretion of such heavy metals, which were within the limits of the Italian Reference Values Society (SIVR), but they did find a positive association between the consumption of crustaceans and seafood and the urinary excretion of arsenic (Soleo et al. 2012).

The aim of this study was to assess the heavy metal exposure in the general population who have no occupational exposure to these metals and who live in the municipalities of Taranto and Statte (the location nearest to the steel factory) and in the municipality of Laterza, 54 km driving distance from Taranto; Laterza is considered as a non-polluted area because no significant industrial plants are present.

## Methods

Between January 2010 and April 2012, a cross-sectional study was conducted to measure the urinary excretion of inorganic arsenic (iAs) and its methylated metabolites monomethyl-arsenic acid (MMA) and dimethylarsinic acid (DMA), chromium (Cr), manganese (Mn), mercury (Hg), and lead (Pb) as well as urinary creatinine, which was used both to confirm the acceptability of urine samples and to adjust the metal concentrations.

The analysis of the urine samples was performed by atomic absorption spectrophotometry (Perkin-Elmer 5100 PC), employing the hydride (arsine) generation technique for determining As, the cold vapors technique for Hg, and the graphite furnace method for Cr, Mn, and Pb according to the NIOSH analytical methods (NIOSH 2003); an automated kinetic Jaffe technique using alkaline picric acid was used to measure creatinine. Urine samples were not processed for metal concentrations if the creatinine excretion was not within the range of 0.3–3.0 g/l (ACGIH 2010).

An internal quality control (IQC), according to the manufacturer’s instructions, was used systematically to verify the reproducibility and the repeatability of the data that were obtained using the standard curve prepared by the operator.

The research was conducted with 350 subjects residing in Taranto and the surrounding area; they were randomly selected from the Regional Assisted Care Registry to reduce the possibility of bias in self-selection. The response rate was high (93.1%).

All of the subjects were contacted in accordance with procedures agreed upon by local general practitioners, who had previously been invited to a dedicated meeting at which they were fully informed about the aims of the study and asked whether they would be willing to collaborate. All subjects agreed to the processing of their personal data and understood that this information was categorized as “sensitive data.” All subjects were informed that the data from the research protocol would be treated in an anonymous and collective way, with scientific methods and for scientific purposes in accordance with the principles of the Helsinki Declaration.

After obtaining informed consent from each participant, qualified health staff administered a standardized structured questionnaire regarding lifestyle habits; they also assessed any confounding factors, including smoking habits, whether they drank tap or bottled mineral water, whether they had eaten seafood a few days before urine collection, whether they had a fireplace inside their home, and whether they used any pesticides or paints. First-void urine (FVU) was sampled from each participant into clean conical 50-ml polypropylene tubes, which were then immediately sealed with O-ring screw caps and packed into coolers with frozen ice packs. Samples were sent to the laboratory and then stored at −20 °C and analyzed within 1 month.

After analysis, we excluded 47 (13.4%) subjects from the study because of creatinine excretion values <0.3 g/l or >3.0 g/l.

**Fig. 1:**
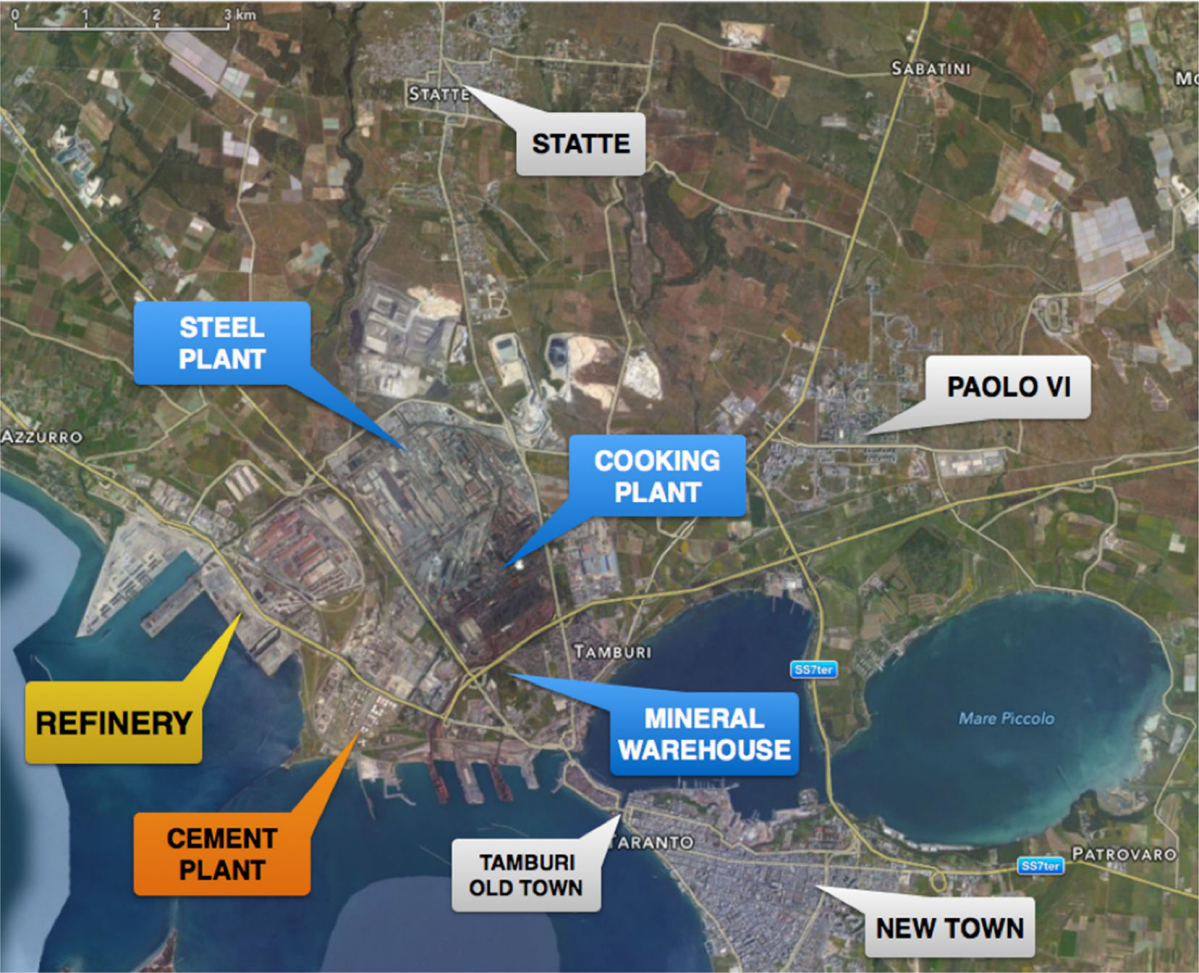
Industrial Taranto area and neighboring area

After these exclusions, the final sample consisted of 279 subjects, including 135 males and 144 females aged between 18 and 77 years (mean age 46.0 ± 13.12 SD). Of the 279 study subjects, 179 resided in the city of Taranto; they were subdivided into three district areas: “Paolo VI” (N 39), “Tamburi–Old Town” (N 50) or “New Town” (N 90). A total of 55 subjects were residents of the nearby Statte municipality (Fig. 1), and 45 resided in the Laterza municipality (Table 1).

**Table 1:** Flying distance from the industrial site

Laterza	34 km
Statte	2.3 km
Paolo VI	3 km
Tamburi–Old Town	200 m
New Town	2.7 km

All samples were registered with a randomly generated identification code to guarantee anonymity. Descriptive analyses of each sample were performed, and the concentrations of the different biological markers studied were measured; all covariates (possible confounders or modifiers of the effect) were taken into account and recorded in the questionnaire. Comparisons among groups were made employing non-parametric techniques (rank sum Wilcoxon-Mann-Whitney test and Kruskal-Wallis test). We also performed a multivariate analysis through a linear regression model, investigating the association of the urinary concentrations of As, Cr, Mn, Hg, and Pb with the explanatory variables obtained by questionnaire. The main variables included in the model were age, sex, body mass index, drinking water, smoking habits, city of residence, dwelling site, the consumption of fish, crustaceans and shellfish in the 48–72 h before collection, the presence of dental fillings, and the use of fireplaces in homes.

A *p* value ≤0.05 was considered significant. Statistical analyses were conducted using packages SAS (v. 9.0) and STATA (v. 11).

## Results

Table 2 shows the urinary levels of iAs + MMA + DMA, Cr, Mn, Hg, and Pb measured in the 279 subjects (135 males and 144 females) residing in Taranto subdivided into three areas: Paolo VI, Tamburi-Old Town, New Town, in Statte and in Laterza (Table 2).

**Table 2:** Median and 95th percentile of the urinary levels of heavy metals (μg/l) in the whole study population

Values	iAs + MMA + DMA	Cr	Mn	Hg	Pb
Mean	6.1	0.5	2.7	1.4	9.3
SD	8.6	0.5	6.7	1.6	6.9
p5	1.4	0.1	0.3	0.2	1.8
p50 (median)	3.8	0.3	1.3	0.8	7.3*
p95	16.8*	1.3*	8.7*	4.5	24.3*
*n*	279	279	279	279	277
SIVR reference values	2.0–15	0.05–0.35	0.2–4.0	0.1–5.0	0.01–2.0

*Statistically significant

### Urinary chromium

The median value was 0.3 μg/l, which was comparable to the upper limit of the range proposed by the SIVR. The 95th percentile was significantly above the reference value limit (Table 2).

The differences in the measured median values among the municipalities were significant, with the highest values found in Statte (Tables 3, 4, 5, and 6). In the different districts of Taranto, the highest median values of urinary concentrations were found in the Paolo VI district (Tables 7, 8, and 9).

**Table 3:** Median and 95th percentile of the urinary levels of heavy metals (μg/l) by municipality in Taranto

Values	iAs + MMA + DMA	Cr	Mn	Hg	Pb
Mean	5.2	0.4	2.4	1.1	9.5
SD	8	0.3	3.9	1.1	6.9
p5	1.5	0.1	0.4	0.2	2.2
p50 (median)	3.8	0.3	1.6	0.7	7.3*
p95	11.1	1*	7.9*	3.9	25.9*
*n*	179	179	179	179	177
SIVR reference values	2.0–15	0.05–0.35	0.2–4.0	0.1–5.0	0.01–2.0

*Statistically significant

**Table 4:** Median and 95th percentile of the urinary levels of heavy metals (μg/l) by municipality in Laterza

Values	iAs + MMA + DMA	Cr	Mn	Hg	Pb
Mean	3.2	0.4	6.2	1.1	4.9
SD	2.3	0.4	14.2	0.8	3.4
p5	0.9	0.1	0.6	0.3	1.2
p50 (median)	2.7	0.3	2.2	0.8	4.1*
p95	8.5	1.2*	22.5*	2.4	12.5*
*n*	45	45	45	45	45
SIVR reference values	2.0–15	0.05–0.35	0.2–4.0	0.1–5.0	0.01–2.0

*Statistically significant

**Table 5:** Median and 95th percentile of the urinary levels of heavy metals (μg/l) by municipality in Statte

Values	iAs + MMA + DMA	Cr	Mn	Hg	Pb
Mean	11.5	0.9	0.7	2.6	12.3
SD	11	1	0.8	2.6	7.3
p5	2.5	0.2	0.2	0.5	2
p50 (median)	8.8	0.5*	0.5	1.8	12.1*
p95	27.1*	2.5*	1.9	7.6*	24.7*
*n*	55	55	55	55	55
SIVR reference values	2.0–15	0.05–0.35	0.2–4.0	0.1–5.0	0.01–2.0

*Statistically significant

**Table 6:** Median and 95th percentile of the urinary levels of heavy metals (μg/l) in the investigated areas

	iAs + MMA + DMA	Cr	Mn	Hg	Pb
Values	p50/p95	p50/p95	p50/p95	p50/p95	p50/p95
TARANTO	3.8/11.1	0.3/1	1.6/7.9	0.7/3.9	7.3/25.9
STATTE	8.8/27.1	0.5/2.5	0.5/1.9	1.8/7.6	12.1/24.7
LATERZA	2.7/8.5	0.3/1.2	2.2/22.5	0.8/2.4	4.1/12.5
SIVR reference values	2.0–15	0.05–0.35	0.2–4.0	0.1–5.0	0.01–2.0

**Table 7:** Median and 95th percentile of the urinary levels of heavy metals (μg/l) in the New Town district of Taranto

Values	iAs + MMA + DMA	Cr	Mn	Hg	Pb
Mean	4.3	0.3	2.6	0.9	9.9
SD	2.4	0.3	4.9	0.8	7.1
p5	1.7	0.1	0.5	0.2	2.5
p50 (median)	3.8	0.3	1.6	0.6	7.5*
p95	9.7	0.9*	4.9*	2.8	24.9*
*n*	90	90	90	90	90
SIVR reference values	2.0–15	0.05–0.35	0.2–4.0	0.1–5.0	0.01–2.0

*Statistically significant

**Table 8:** Median and 95th percentile of the urinary levels of heavy metals (μg/l) in the Paolo VI district of Taranto

Values	iAs + MMA + DMA	Cr	Mn	Hg	Pb
Mean	4	0.4	2.6	0.9	7.2
SD	3.7	0.2	2.1	0.8	3.4
p5	1.4	0.1	0.5	0.2	2.1
p50 (median)	2.7	0.4*	2.2	0.8	7*
p95	9.1	0.8*	8.7*	1.4	13.6*
*n*	39	39	39	39	38
SIVR reference values	2.0–15	0.05–0.35	0.2–4.0	0.1–5.0	0.01–2.0

*Statistically significant

**Table 9:** Median and 95th percentile of the urinary levels of heavy metals (μg/l) in the Tamburi district of Taranto

Values	iAs + MMA + DMA	Cr	Mn	Hg	Pb
Mean	7.8	0.4	1.8	1.6	10.5
SD	14.3	0.4	2.8	1.6	8.1
p5	1.4	0.1	0.2	0.3	2
p50 (median)	4.6	0.3	0.8	0.9	7.3*
p95	14.3	1.2*	8.6*	4.5	28.7*
*n*	50	50	50	50	49
SIVR reference values	2.0–15	0.05–0.35	0.2–4.0	0.1–5.0	0.01–2.0

*Statistically significant

The median values of urinary chromium were comparable in both sexes, in all age classes, among smokers and non-smokers and regardless of whether they drank tap or bottled mineral water (Table 10).

**Table 10:** Urinary excretion of heavy metals (μg/l) in relation to the variables listed

	Sex		Smoking habit		Water		Seafood consumption	
	Male	Female	Yes	No	Tap	Bottled	Yes	No
iAs + MMA + DMA	4.1	3.8	4.1*	3.8	3.6*	2.5	9.8*	3.8
Cr	0.4	0.3	0.5	0.3	0.5	0.4	0.2	0.1
Mn	1.2	1.4*	1.7	1.2	0.6	1.7*	1.0*	0.9
Hg	1*	0.8	1.1	0.8	1.0	0.9	1.7	0.8
Pb	7.3	7.4	7.7	7.2	7.3	6.9	7.8*	7

*Statistically significant

The multivariate analysis showed the following results. For Cr, we found an association with the city of residence; specifically, we found a higher concentration in the people living in Statte vs Taranto (*p* = 0.001).

### Urinary lead

The median urinary concentration value was significantly higher than the Italian reference value; the 95th percentile value was 12 times higher than the upper limit value of 2 μg/l (Table 2). They also exceeded the upper limit of the SIVR range. The median urinary concentrations by municipality were significantly higher in Statte, in the 95th percentile (Tables 3, 4, 5, and 6).

Statistically significant differences were found among the municipalities (Tables 3, 4, 5, and 6), with the highest value (12.1 μg/l) in Statte and in those who had eaten seafood (Table 10) 48–72 h before urine collection (7.8 vs 7.0 μg/l). There were no significant differences according to sex, age class, or drinking water (Table 10).

For Pb, the multivariate analysis found an association with the consumption of fish and crustaceans in the 48–72 h before collection (*p* = 0.041) and with city of residence; specifically, we found a lower concentration in the people living in Laterza vs Taranto (*p* < 0.001).

### Urinary inorganic arsenic and methylated metabolites

The median urinary concentration in the entire study population was within the SIVR reference limit, whereas the 95th percentile was higher than the upper limit (Table 2). Considering the municipalities, the 95th percentile was higher than the reference value only in Statte (Table 5). Moreover, the median urinary concentration of iAs + MMA + DMA was significantly higher in Statte than in Taranto or Laterza, although it was still within the range limits (Tables 3, 4, 5, and 6). In the different districts in the city of Taranto, the 95th percentile and median urinary values remained within range limits (Tables 7, 8, and 9).

When analyzing the urinary excretion of iAs + MMA + DMA in relation to the variables considered in the study population, similar median values were obtained in both sexes. In subjects who drank tap water, urinary iAs + MMA + DMA values (3.6 μg/l) were higher than in those who drank bottled mineral water (2.5 μg/l). Slightly higher values were found in smokers (4.1 μg/l) than in non-smokers (3.8 μg/l). Statistically significant differences were found when comparing the urinary concentrations in those who had eaten shellfish and/or seafood in the 48–72 h before sampling (9.8 vs 3.8 μg/l) (Table 10).

The multivariate analysis showed the following results. For As, we found an association with the city of residence; specifically, we found a higher concentration in the people living in Statte vs Taranto (*p* = 0.001) and a lower concentration in the people living in Laterza vs Taranto (*p* = 0.037) with the consumption of crustaceans in the 48–72 h before collection (*p* = 0.019).

### Urinary mercury

The median urinary concentrations were within the reference values suggested by the SIVR both for the entire study population (Table 2) and when analyzing the Taranto districts separately (Tables 7, 8, and 9). The 95th percentile of the urinary concentrations in the population of residents of Statte was higher than the SIVR reference value (Table 5). The differences between the municipalities were statistically significant (Tables 3, 4, 5, and 6), with the highest median value in Statte. The 95th percentile of urinary excretion of Hg was significantly higher in the Tamburi-Old Town district than in Paolo VI or New Town (Tables 7, 8, and 9). Men showed higher values (1.0 μg/l) than women (0.8 μg/l). Higher values were obtained in smokers compared to non-smokers (1.1 vs 0.8 μg/l), and a statistically significant difference was found in those who had eaten seafood products in the 48–72 h before sampling (1.7 vs 0.8 μg/l) (Table 10).

For Hg, the multivariate analysis found an association with the city of residence; specifically, we found a higher concentration in the people living in Statte vs Taranto (*p* = 0.001) with sex—women show a lower concentration than men (*p* = 0.028)—and with the consumption of crustaceans in the 48–72 h before collection (*p* = 0.019).

### Urinary manganese

The median values were within the reference limits proposed by the SIVR, while overall, the 95th percentile of urinary concentrations was higher than the SIVR upper limit (Table 2). There were statistically significant differences among the municipalities, but they all remained within the reference values (Tables 3, 4, 5, and 6). In Taranto, there were significantly higher values in the Paolo VI district than in Tamburi and New Town (Tables 7, 8, and 9), although they did not exceed the upper limit value of 4 μg/l.

There were slightly higher values in women compared to men (1.4 vs 1.2 μg/l), in smokers compared to non-smokers (1.7 vs 1.2 μg/l), and in those who drank bottled mineral water compared to those who drank tap water (1.7 vs 0.6 μg/l) (Table 10).

The multivariate analysis showed the following results. For Mn, we found an association with the city of residence; specifically, we found a lower concentration in the people living in Statte vs Taranto (*p* = 0.027).

Table 6 shows the urinary levels of iAs + MMA + DMA, Cr, Mn, Hg, and Pb measured in the whole study population subdivided into the three areas of Taranto, Statte, and Laterza (Table 6).

## Discussion

To date, few studies have investigated exposure to heavy metals in a population residing in a high risk environmental area such as Taranto, Apulia Region (Southern Italy). Our study, conducted in a sample of approximately 300 residents of Taranto and the surrounding area, used biological monitoring techniques, and showed high urinary concentrations of nearly all of the heavy metals investigated, especially lead and chromium.

The median value for chromium (0.3 μg/l) was the upper limit value of the relative SIVR range, while the 95th percentile was actually higher than the proposed SIVR upper limit. There were no significant differences in urinary excretion by sex, age, or drinking water, unlike in other reports in literature, in which an association was found between residing in an industrial area, the number of cigarettes smoked and the type of water drunk (EPA 1984; SIVR 2011; Zhitkovich 2005). Tobacco smoke is known to contain chromium (VI), and indoor air polluted by cigarette smoke can contain hundreds of times the amount of chromium (VI) found in outdoor air (IARC 2012a, b).

Drinking water is another known source of non-occupational exposure to chromium. The chromium concentration limit in drinking water applied both in Italy (Legislative Decree no. 31/2001) and in the USA (U.S. Environmental Protection Agency—EPA) is 50 μg/l (ATSDR 2000). However, in a study conducted in California (USA), 38% of municipal sources of drinking water reportedly showed higher levels of chromium (VI) than the detection limit of 1 μg/l (Sedman et al. 2006).

The first epidemiological study to probe the effects of environmental exposure to chromium (VI), known to be the carcinogenic form of this metal, dates back to 1980 in Sweden. It was conducted in populations residing in the vicinity of two Swedish steel foundries producing iron and chromium alloys. The mortality rate for lung cancer in the area was higher than expected for the general population in the region (Axelsson and Rylander 1980).

For lead, the median value and the 95th percentile observed in the present study population were significantly higher than the upper limit of the reference values range proposed by the SIVR. The multivariate analysis found an association with consumption of fish and crustaceans and with the city of residence; specifically, we found a lower concentration in the people living in Laterza vs Taranto. Various studies have investigated the role of fresh and/or canned seafood on the levels of lead absorbed (Canli and Atli 2003; Eboh et al. 2006; Hosseini et al. 2015; Turkmen et al. 2008). Some monitoring studies of the Adriatic Sea have shown that the maximum levels exceeded the limits not only for lead but also for cadmium and mercury (Storelli et al. 2003; Storelli and Marcotrigiano 2001).

Lead is widely used in various paints because of its anticorrosive properties and its ability to hold pigments together. Mohammed et al. demonstrated that painters have higher blood lead levels than controls (Mohammad et al. 2008), which is consistent with previous reports on blood lead levels in Indian painters (Rao et al. 2006; Patil et al. 2007).

In our study, no association between the use of paints and the urinary concentrations of Pb was found. We also studied the levels of urinary lead in relation to whether the subjects drank tap water or bottled mineral water. Pb was widely used in the past in the main supply pipes in various European states, including Italy. Now, however, the use of Pb in materials used to convey water destined for human consumption is subject to rigorous regulations to limit the risks of water contamination.

A research project supported by the Italian Ministry of Health and conducted by the Italian Health Institute collected data over the period 2002–2004 in 21 cities. Lead concentrations in tap water that exceeded the limit value of 10 μg/l were found in approximately 2–4% of the samples. Higher rates of non-conformity to the regulations were shown in samples collected after 4 h of stagnation in the main supply network (Veschetti et al. 2009).

Higher median levels of urinary Pb were found in the smokers in our study. Cigarette smoke is well known to be an important source of exposure to lead, which can accumulate in the body tissues and fluids (Galazyn-Sidorczuk et al. 2008).

The multivariate analysis showed an association with the city of residence and with consumption of crustaceans. Consistent with data in the literature, the subjects who had eaten seafood and/or shellfish 48–72 h before urine collection had higher levels of urinary excretion of arsenic (9.8 vs 3.8 μg/l). In fact, diet is the main source of non-occupational exposure to arsenic (Vimercati et al. 2009). Foods with the highest content of arsenic include some marine organisms, such as shellfish and crustaceans (Argese et al. 2005; Fattorini et al. 2004; Lopez et al. 1994; WHO 2001). However, some authors did not find a positive association between the consumption of seafood and/or shellfish and the urinary concentrations of As. In particular, Hsueh et al. 2002 found no differences in the urinary concentrations of the various As species before and after refraining from eating seafood for 3 days.

Moreover, despite the notorious toxic effects of arsenic in humans, it is still used in both agriculture and industry (Park et al. 2010; Kumaresan and Riyazuddin 2001). Arsenic-based pesticides are the best examples of agricultural applications of arsenic. This large amount of inorganic arsenic-based pesticide has led to serious environmental arsenic contamination (Datta and Sarkar 2005).

Given the notorious adverse effects of arsenic exposure in humans, the US Environmental Protection Agency (EPA) banned the use of many inorganic arsenic-based pesticides during the late 1980s and early 1990s (Quazi et al. 2013).

Although some countries have issued documents to phase out organo-arsenical pesticides from the market, large agricultural sites contaminated by years of organo-arsenical pesticide application still exist. These agricultural lands might pose significant health risks in the present and in the future (Li et al. 2016).

In our study, no association was found between the use of pesticides and urinary concentrations of As. There were significant differences between those who drank tap water and those who habitually drank bottled mineral water. The contamination of the main water supply remains a major source of exposure to inorganic As in many parts of the world (IARC 2004), despite the fact that in 1993, the WHO recommended that levels of As in drinking water should not exceed 10 μg/l (WHO 1993). On the other hand, a recent analysis of 40 different labels of bottled mineral water on sale in Italy demonstrated higher levels of total As than the legal limit in five of them (Signorile et al. 2007). In contrast, in the surveys of water in the Apulian aqueduct over the period 2004–2006, total As values were consistently below 1 μg/l.

We also found a difference in the urinary excretion of As between smokers and non-smokers. According to the WHO, the arsenic content in mainstream cigarette smoke is in the range of 40–120 ng per cigarette, and the daily intake of arsenic for those who smoke 20 cigarettes a day is 0.8–2.4 μg.

Unexpectedly, the highest median levels of urinary manganese were found in subjects residing in Laterza, widely within the upper limit value, and in those who drank bottled mineral water. The multivariate analysis showed an association with city of residence; specifically, we found a lower concentration in the people living in Statte vs Taranto.

The results of some epidemiological studies have also established a correlation between the exposure to this metal through the drinking water and the onset of some neurological alterations (Bouchard et al. 2007; Bouchard et al. 2011; Ericson et al. 2007; Khan et al. 2011; Kim et al. 2009; Menezes-Filho et al. 2009; Takser et al. 2003; Wasserman et al. 2006; Wright et al. 2006; Storelli and Marcotrigiano, 2005). The 95th percentile of the urinary concentrations was higher overall than the upper limit of the SIVR range.

The urinary concentrations of mercury were comparable to the SIVR reference values in the entire study population and were higher in Statte than in Taranto or Laterza. Indeed, the 95th percentile of the urinary concentration was above the SIVR reference range for the population residing in Statte.

As with As and Hg, and to a lesser extent for Pb, eating seafood and shellfish affects the urinary excretion values of these metals, which is why these variables were taken into account when interpreting the results. In fact, non-occupational exposure to mercury is known to be mainly through the dietary intake of shellfish and seafood. Approximately 95% of the Hg present in seafood is in the form of methylmercury, which is a toxic form of the metal.

Studies of seafood fished in the Ionian Sea to investigate the levels of Hg, Cd, and Pb showed high levels of only Hg, ranging from 0.31 to 1.50 μg/g in fresh seafood (Bouchard et al. 2007). In our study, and in the literature, there were significantly higher levels of Hg in subjects who had eaten seafood in the 48–72 h before urine collection. The multivariate analysis found an association with sex, with women having a lower concentration than men with consumption of crustaceans, and with city of residence; specifically, we found a higher concentration in the people living in Statte vs Taranto.

Moriske et al. found higher concentrations of heavy metals in indoor air pollution in houses with coal burning and open fireplaces than in homes with central heating (Moriske et al. 1996). In our study, we investigated the association between having a fireplace in the home and the urinary excretion of heavy metals, but did not find any association.

Overall, the biological monitoring data reveal the existence of environmental pollution by heavy metals in the Taranto area and, specifically, in the Statte municipality. This finding could be associated with the presence of industrial plants and, in particular, with the integrated cycle steel foundry, even though the Mn concentrations, which could be a marker of the foundry emissions, were actually higher in Laterza than in the Statte of Taranto.

However, in our study, it was not possible to correlate the biological monitoring data with the environmental data because the information collected by the official institutions and/or those in the literature were incomplete and only provided by European Monitoring and Evaluation Programme (EMEP). For the whole province of Taranto, the value of emissions of Pb into the atmosphere in 2009 was 38 t, one the highest in Europe, and the emissions of Hg in the same year was 510 Kg. There are no available data concerning the emissions of the other metals. In the future, therefore, we believe it will be necessary to carry out an organized environmental monitoring program, taking into consideration all exposure routes to correlate the environmental concentrations of these metals with the biomonitoring results.

In any case, the data we obtained, which may be further confirmed by larger population studies, are sufficient to warrant the expectation that local and national institutions should be required to adopt preventive measures to reduce the environmental exposure of the general population to heavy metals, especially lead and chromium. Such actions could help to reduce the health risks, including those of a carcinogenic nature, posed to populations residing in areas with a known high environmental impact.

## Conclusions

We conducted a study to evaluate the exposure to heavy metals in the industrial city of Taranto and the surrounding area in Southern Italy through biological monitoring techniques. We measured the levels of inorganic arsenic and methylated metabolites, lead, cadmium, chromium, and manganese in the urine samples of 279 subjects residing in Taranto and neighboring areas. Our study results showed high urinary concentrations of nearly all of the heavy metals investigated. This finding could be related to the presence of industrial plants and is sufficient to warrant the expectation that local and national institutions should be required to adopt preventive measures to reduce the environmental exposure of the general population to heavy metals.

Further epidemiological studies with larger samples and including environmental air quality data will be necessary to confirm our results.
